# Acupuncture for Treating Whiplash Associated Disorder: A Systematic Review of Randomised Clinical Trials

**DOI:** 10.1155/2014/870271

**Published:** 2014-05-08

**Authors:** Tae-Woong Moon, Paul Posadzki, Tae-Young Choi, Tae-Yong Park, Hye-Jung Kim, Myeong Soo Lee, Edzard Ernst

**Affiliations:** ^1^Medical Research Division, Korea Institute of Oriental Medicine, Daejeon 305-811, Republic of Korea; ^2^Jaseng Oriental Hospital, Bundang 463-824, Republic of Korea; ^3^The Centre for Public Health, Liverpool John Moores University, Liverpool L3 2AY, UK; ^4^Department of Oriental Medicine, International St. Mary's Hospital, Incheon 404-834, Republic of Korea; ^5^Department of Family Medicine, International St. Mary's Hospital, Incheon 404-834, Republic of Korea; ^6^Complementary Medicine, Peninsula Medical School, University of Exeter, Exeter EX2 4SG, UK

## Abstract

The aim of this systematic review was to determine the effectiveness of acupuncture for the treatment of whiplash associated disorder (WAD). Twenty databases were searched from their inceptions to Oct. 2013. Randomised clinical trials (RCTs) of acupuncture (AT), electroacupuncture (EA), or dry needling (DN) for the treatment of WAD were considered eligible. The risk of bias was assessed using the Cochrane tool. Six RCTs met the inclusion criteria. Most of the included RCTs have serious methodological flaws. Four of the RCTs showed effectiveness of AT, AT in addition to usual care (UC), AT in addition to herbal medicine (HM) or EA was more effective than relaxation, sham EA, sham EA in addition to HM or UC for conditioned pain modulation (CPM) and alleviating pain. In one RCT, DN in addition to physiotherapy (PT) had no effect compared to sham-DN in addition to PT for the reduction of pain. None of the RCTs showed that AT/EA/DN was more effective than various types of control groups in reducing disability/function. One RCT did not report between-group comparisons of any outcome measures. The evidence for the effectiveness of AT/EA/DN for the treatment of WAD is limited. Therefore, more research in this area is warranted.

## 1. Introduction

Whiplash injury or whiplash associated disorder (WAD) is defined as a bony or soft tissue injury to the neck following an acceleration-deceleration mechanism of energy transfer resulting predominantly from motor vehicle accidents [1]. WAD is a common cause of chronic disability and is associated with a wide variety of clinical manifestations, including chronic neck pain and stiffness, headache, vertigo, dizziness, paresthesia, dysphasia, psychological distress, anxiety, depression, concentration and memory difficulties, sleep disturbances, or social isolation [2]. WAD is a serious, global healthcare issue; it accounts for as much as 83% of motor vehicle collision injuries [3]. In the United Kingdom, the incidence of WAD accounted for 57% of all emergency cases in 1995 and 2000 [4]. It is estimated that the incidence of whiplash is approximately 4 per 1,000 persons [2], although these rates may vary across the globe. It is also a costly problem. The annual costs associated with the management of WAD and associated with time off work are approximately $3.9 billion in the USA and 10 billion EUR in Europe [5]. The therapeutic management of WAD may involve multiple interventions, including but not limited to physiotherapy (PT), occupational therapy, psychotherapy, and educational approaches [5].

Acupuncture therapy (AT) is one of most popular types of complementary and alternative medicine (CAM). It is now a widely accepted treatment for a variety of conditions, many of which are associated with pain [6, 7]. AT is defined as the insertion of needles into the skin and underlying tissues at particular sites, known as acupoints, for therapeutic or preventive purposes [8]. AT includes electroacupuncture (EA), heat (including moxibustion), pressure, and laser-generated light. EA is defined as a “therapy derived from Chinese acupuncture but using modern electronics” [9]. Dry needling (DN) and AT, while using the same needle types, are two very different treatments. DN is defined as “a technique that uses needles to treat myofascial pain in any body part, including the low-back region” [10]. All three techniques are sometimes utilised as symptomatic treatments of WAD, but their effectiveness remains unknown [11, 12].

Whiplash often leads to significant chronic pain and it is useful to refer to the current evidence regarding acupuncture applied to this condition. There are several related systematic reviews of acupuncture for neck pain [13–18]. One such review included 5 RCTs and analysis in the form of individual patient data [17]. This review suggested that acupuncture may be beneficial for pain relief, although the review analysed nonspecific neck and low back pain together. Recently, another review was published in 2012 that was based on 22 RCTs [15]. This review failed to show any beneficial effects of acupuncture for several types of neck pain. Other reviews are outdated [13, 14, 16, 18]. Currently available guidelines for the management of neck pain or WAD do not include acupuncture as a treatment [19, 20] or include it only in combination with manual and physical therapies and exercise [21, 22]. To the best of our knowledge, there are no systematic reviews (SRs) of AT/EA/DN for the treatment of WAD. This SR aims to critically evaluate all randomised clinical trials (RCTs) of AT or EA or ND compared with various controls for the treatment of patients with WAD.

## 2. Methods

### 2.1. Data Source

The recent Preferred Reporting Items for Systematic Reviews and Meta Analyses (PRISMA) statement was used for the reporting structure of this systematic review. The following 20 databases were searched from their inception to Oct 2013: AMED, CINAHL, http://www.clinicaltrials.gov, EMBASE, ISI Web of Knowledge, MEDLINE, PEDro, PSYCINFO, Rehab Trials, Rehadat, The Cochrane Library, one Chinese database (China National Knowledge Infrastructure), three Japanese databases (J stage, Journal archive, and Science Links Japan), and five Korean databases (Korea Institute of Science and Technology Information, DBpia, Korea National Assembly Library, Korean Studies Information Service System, and Oriental Medicine Advanced Searching Integrated System). Details of the search strategy for MEDLINE are available in the appendix. The same search terms were used in Korean, Chinese, and Japanese. Authors (*N* = 3) were also contacted and asked for any unpublished data. In addition, the reference lists of all located articles were hand-searched for further relevant literature. Hard copies of all included articles were read in full.

### 2.2. Search Strategy

The detailed search strategy for MEDLINE was as follows.

1. Acupuncture therapy/OR electroacupuncture/OR (acupunct$ or electroacupunct$ or electroacupunct$).mp./dry needl$.
2. Whiplash OR whiplash injur$ OR whiplash associated disorder$ OR neck sprain OR neck inur$.
3. (randomised controlled trial).pt. OR (clin$ adj5 trial$).ti,ab. OR ((singl$ or doubl$ or tripl$ or trebl$) adj5 (blind$ or mask$ or sham)).ti,ab OR random$.ti,ab OR control$.ti,ab. OR prospectiv$.ti,ab. OR exp clinical trial/OR follow-up studies/or prospective studies/OR double-blind method/or random allocation/or single-blind method/OR exp Research Design/.
4. 1 AND 2 AND 3.

### 2.3. Study Selection

#### 2.3.1. Type of Studies

This review included RCTs that assessed the effect of AT on whiplash injury, regardless of the type of reporting, language, or blinding.

#### 2.3.2. Type of Participants

This study included patients with WAD by any defined or specified diagnostic criteria, regardless of sex, age, or race. Studies in which patients suffered from any type of ailment, such as muscular or psychological problems due to whiplash injury, were included.

#### 2.3.3. Types of Interventions

Studies that evaluated any type of AT were included. Treatments involving needle insertion at acupoints, pain points, or trigger points were described as AT. EA was also included. DN, the more common and best supported approach, targets myofascial trigger points. Trials testing other forms of AT, such as laser AT, herbal AT, moxibustion, acupressure, pressed studs, or transcutaneous electrical stimulation, were excluded. Control interventions (in controlled studies) included treatments such as usual care, sham treatment (interventions mimicking “true” AT/true treatment but deviating in at least one aspect considered important by AT theory, such as skin penetration or correct point location), or other treatment (e.g., relaxation, physiotherapy). We also included trials that compared AT plus another active treatment versus the other active treatment alone. Thus, we included all pragmatic trials that compared AT with any other treatments (e.g., drugs, exercise, etc.). Because our objective was to evaluate the effects of AT compared to non-AT controls, we excluded RCTs in which one form of AT was compared to another form of AT.

#### 2.3.4. Outcome Measures

Outcome measures pertaining to pain intensity, quality of life, and function were collected and assessed. Some outcome measures, such as the visual analogue scale (VAS), verbal scale, neck disability index (NDI), short-form 36 (SF-36), and range of movement (ROM), were anticipated based on previous analyses.

### 2.4. Data Extraction and Risk of Bias Assessment

The data screening and selection were conducted by two independent reviewers (T.-W. Moon and P. Posadzki) and were verified and validated by the third author (M.S. Lee). Two authors (T.-W. Moon and P. Posadzki) extracted the data using a predefined data extraction form. The Cochrane tool was used to assess the quality of the RCTs. Two authors (T.-W. Moon and P. Posadzki) independently assessed the risk of bias of the included studies [29]. Disagreements were settled through a joint discussion between the authors.

### 2.5. Data Synthesis

The mean changes in pain intensity, quality of life, and function compared with baseline were defined as the primary outcome measures, and the differences between the intervention groups and the control groups were assessed. The effect size for each individual outcome variable was estimated and we planned to combine the data from the individual studies for a meta-analysis (for studies with little heterogeneity). Continuous data were presented as the mean differences (MD), and dichotomous data were presented as the relative risks (RR) with 95% confidence intervals (CIs). In cases of outcome variables with different scales, the standard mean difference (SMD) was used instead of the weighted MD (WMD). If the meta-analyses exhibited heterogeneity (defined as results of tests of heterogeneity indicating that *P* < 0.1 and *I* ^2^ ≥ 50%), a random effects model was used to assess the combined efficacy values; otherwise, fixed effects models were used for these assessments. If more than 10 studies were found to conduct meaningful assessments of publication bias, funnel plots were used. The Review Manager 5.1 software (Copenhagen: The Nordic Cochrane Centre, The Cochrane Collaboration, 2011) was used for statistical analyses.

## 3. Results

### 3.1. Study Selection

**Figure 1:**
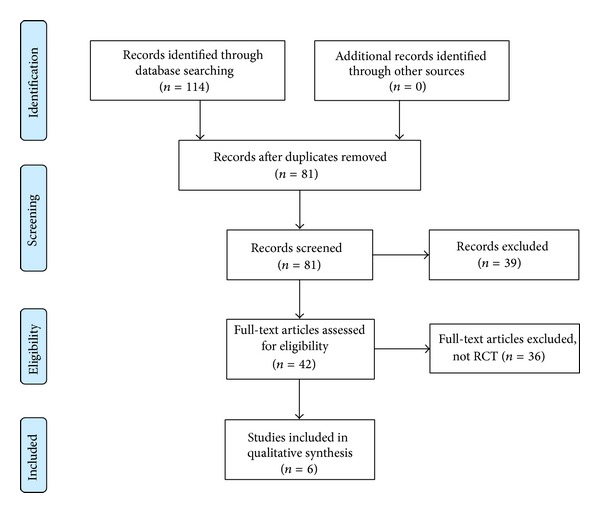
PRISMA diagram for the included studies. RCT: randomised clinical trial.

The searches generated 114 articles, and 108 were excluded (Figure 1). Six RCTs met our inclusion criteria. The key data from the included studies are summarised in Table 1 [23–28]. The trials originated from Australia [25], Austria [28], Belgium [24], Korea [23, 26], and the UK [27]. Three RCTs [23, 24, 28] used AT; two [25, 26] used EA; and the remaining study used DN [27]. A total of 348 patients were included in the analyses.  Table 2 summarises details of each treatment regimen.

A total of 309 patients were involved in these 5 studies. In two RCTs [25, 28], patients with WAD grade I or II were recruited, and in one RCT [27], patients with WAD grade two were recruited. The other RCTs [23, 26] did not mention WAD-grading.

Pain intensity was analysed by the VAS [23, 25, 26], short-form McGill pain questionnaire (SF-MPQ) [27], and modified brief pain inventory short-form (m-BPI-sf) [27]. The short-form 36 (SF36) was used to analyse quality of life, and the ROM [28] and NDI [25–27] were used to assess function.

### 3.2. Assessment of Risk of Bias (ROB)

**Figure 2:**
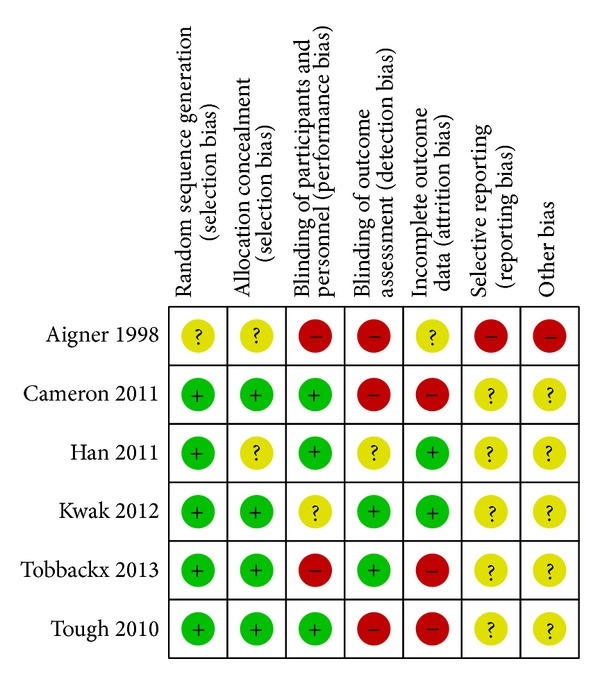
Risk of bias summary: review authors' judgments about each item's risk of bias for each included study. +: low risk of bias; −: high risk of bias; ?: unclear.

**Figure 3:**
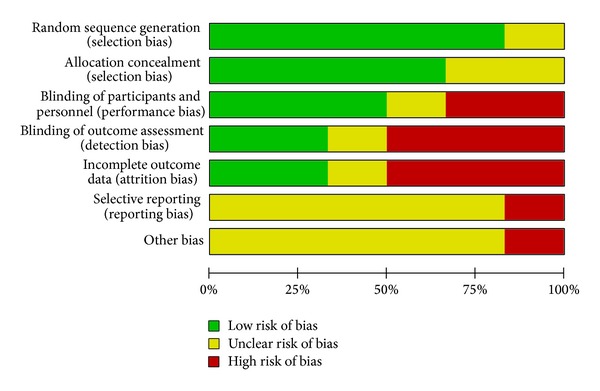
Risk of bias graph: review authors' judgments about each item's risk of bias presented as percentages across all included studies.

The results of ROB were shown in Figures 2 and 3. Five RCTs [23–27] had a low ROB with regards to adequate sequence generation and addressing incomplete data, and one RCT [28] had an unclear ROB in both dimensions. With regard to allocation concealment, four RCTs [23–25, 27] had a low ROB and two RCTs [26, 28] had unclear ROBs. With regards to participant and personnel blinding, three RCTs [25–27] had a low ROB; two [23, 24] had an unclear ROB; and one had a high ROB [28]. With regard to assessor blinding, two RCTs [23, 24] had a low ROB, two [27, 28] had a high ROB, and the remaining two [25, 26] had an uncertain ROB. All six RCTs had low ROBs in selective outcome reporting. All but one RCT had high ROBs in other sources of bias [28].

### 3.3. Study Characteristics

Kwak et al. [23] tested the effectiveness of 6 sessions of AT plus usual care (UC) compared with UC alone in 40 patients with WAD. At the two week follow-up, the authors reported significant reductions in pain intensity (VAS) in the treatment group (*P* < 0.001; MD = −1.85; 95%CI −2.67 to −1.02) compared with controls (*P* < 0.001; MD = −0.40; 95% CI −1.18 to 0.38) and concluded that AT was associated with a significant alleviation of pain.

Tobbackx et al. [24] compared the effect of one session of AT with relaxation in 38 patients with WAD grades I-III. The authors reported significant reductions in pressure pain sensitivity (PPS) and PPS during CPM (both at *P* < 0.001; no CIs) following AT compared with the controls and concluded that one session of AT results in acute improvements in PPS in the neck and calf of patients with chronic WAD; AT had no effect on CPM or the temporal summation of pressure pain.

Cameron et al. [25] investigated the effectiveness of 12 sessions of EA compared with sham EA in 116 patients with WAD grade I or II. The authors reported significant reductions in pain intensity (VAS) at the three- (*P* = 0.05; 95% CI −1.0 to −0.3) and six-month (*P* = 0.007; 95% CI −1.2 to −0.l) follow-ups in the EA group compared with controls; the authors found no significant reductions in NDI or SF-36 at three or 6 months. They concluded that real EA was associated with a significant reduction in pain intensity, albeit clinically insignificant; and there were no changes in disability or quality of life.

Han et al. [26] compared the effect of 8 sessions of EA plus Wuji-san (a Chinese herbal mixture) with that of sham EA plus the mixture in 58 WAD patients. At the four-week follow-up, the authors reported a significant reduction in pain intensity (VAS) compared with controls (*P* = 0.043; no CIs) and no significant reduction in disability. The authors concluded that concomitant treatment with EA could be recommended as a useful therapy for WAD patients.

Tough et al. [27] tested the feasibility of a phase III RCT of DN in addition to physiotherapy (PT) (unknown number of sessions) versus sham DN in addition to PT in 34 females with grade II WAD. After six weeks, the authors reported no between-group differences in SF-MPQ (*P* = 0.67; no CIs), m-BPI-sf (*P* = 0.56; no CIs), or NDI (*P* = 0.43; no CIs) and concluded that a large RCT is both feasible and clinically relevant.

Aigner et al. compared the effect of AT (unknown number of sessions) with that of PT plus drugs (chlormezanone and paracetamol) in 61 patients with grades I-II WAD. At the eight- and twelve-month follow-ups, the authors reported increased ROM and decreased duration of acute complaints and drug intake following AT. However, no statistical tests were performed between the two groups.

### 3.4. Methods of Sham Intervention

Three RCTs [23, 25, 27] used sham intervention as a control. One RCT [25] penetrated nonacupoints without electronic stimulation. The other RCT [26] penetrated the same acupoints as the real EA without electrical stimulation. A third RCT used nonpenetrating DN [27].

### 3.5. Adverse Events

Adverse events were mentioned in three studies [23, 25, 27], but no serious adverse events were reported. Most of the reported mild adverse events occurring with AT were bruising, fatigue, slight pain, sweating, and low blood pressure.

## 4. Discussion

To the best of our knowledge, this is the first SR of RCTs on the effectiveness of AT/EA/DN for the treatment of WAD. Only 6 RCTs exist, and 4 of them suggest that AT and/or EA have a positive effect on pain in WAD patients. However, none of them showed effectiveness in reducing disability. The evidence from these RCTs of the use of AT/EA for the treatment of WAD is, thus, ambiguous and inconclusive for several reasons.

Our SR reveals a paucity of large RCTs and some weaknesses in most of them. For instance, only two [23, 24] RCTs used assessor blinding. Three RCTs [25–27] controlled for placebo effects by performing sham techniques. Three RCTs failed to perform power and sample size calculations [23, 26, 28]. One RCT [28] did not use any statistical tests, which left its conclusions open to criticism. The other study [25] was unable to show clinically meaningful improvements in pain. The effect size of AT/EA ranged from −0.03 (small) [27] to 0.8 (large) [26] (mean = 0.34-small). In 2 of the 6 RCTs, the statistics needed for effect size calculations were not reported [23, 28].

Of the six RCTs, three RCTs were patient blinded [25–27] and two studies were assessor blinded [23, 24]. Two RCTs were self-reported subjective questionnaires completed by patients [25, 27]. The concealment of treatment allocation was reported in four trials [23–25, 27]. Trials with inadequate blinding and inadequate allocation concealment are likely to show exaggerated treatment effects and, thus, may not be reliable [30].

One problem with clinical trials of AT is finding a suitable placebo control [31]. Several sham AT methods include puncturing the skin outside acupoints, inserting needles on nonacupoints, or superficially puncturing the skin without stimulation. However, there is currently no evidence of the superiority of real AT compared with sham AT, regardless of the AT technique used [32]. Therefore, a range of methods have been used, and some methods may not be adequate. Another problem is blinding. A study of AT is challenging to conduct because it is almost impossible to blind acupuncturists to the treatments they are delivering. Furthermore, it may be difficult to convince the study participants that sham AT is a credible treatment.

The included studies were heterogeneous in terms of methodological design, WAD grades, control groups, and primary outcome measures. Specifically, the types of trials included waiting-list controlled [23], cross-over [24], placebo-controlled [25–27], and parallel groups [28]. The WAD grades ranged in each study: I or II [25, 28], II [27], and I–III [24]. The control groups were drugs plus PT [28], relaxation [24], sham-AT plus PT [27], sham EA [25, 26], and UC [23]. The primary outcome measures were CPM [24], HAD-A [27], drug intake, duration of acute complaints [28], NDI [25–27], m-BPI-sf [27], ROM [28], SF-MPQ [27], SF-36 [25], and VAS [23, 25, 26]. The frequency of AT/EA sessions ranged from 1 [24] to 12 [25]. The total number of acupoints treated ranged from two [28] to 20 [23].

The importance of the Standard for Reporting Interventions in Controlled Trials of Acupuncture (STRICTA) guidelines [33] has recently been emphasised [34]. Unfortunately, none of the RCTs fully described the details of their AT/EA/DN treatments, making them difficult or even impossible to reproduce. Two RCTs failed to mention the total number of AT sessions [27, 28]. One RCT [27] failed to provide details of the myofascial trigger points needled. Two RCTs [23, 26] did not mention WAD grades. AEs associated with the use of AT for treating the symptoms of whiplash injury are usually mild and do not appear to require acute medical attention or hospitalisation. Three RCTs [24, 26, 28] failed to report the incidence of AEs. The STRICTA guidelines and medical ethics require the reporting of AEs. Unless future trials of AT or EA follow STRICTA, they will contribute little to the evidence base.

WAD not only causes musculoskeletal strain but also leads to a wide variety of psychological and social disorders [35, 36]. Thus, addressing AT's mechanism of action in WAD patients might pose considerable challenges. Apart from nonspecific psychological (placebo) effects related to the patient's belief that treatment will be effective, the antinociceptive effect of needling might involve a reduction of inflammatory pain and proinflammatory cytokines through the activation of endogenous cannabinoids and peripheral cannabinoid receptors [37, 38].

Our review has several limitations that should be kept in mind when interpreting its results. First, although we searched an extensive number of databases, we cannot be sure that all relevant articles were found. In particular, RCTs from Asian countries may have used indexing terms other than whiplash and, therefore, would not have been identified in our search. Second, due to the statistical and clinical heterogeneity of the studies, a formal meta-analysis was deemed implausible. Third, the total number of trials included in our analyses and the total sample size are too small to allow definitive judgments. Finally, publication bias may have negative studies. The present review has strengths, including a thorough search strategy without language restrictions and a critical appraisal of the included trials.

In conclusion, the evidence for the effectiveness of AT for WAD is limited. More research is warranted in this area.

## Figures and Tables

**Table 1:** Key data for randomised clinical trials on acupuncture for treating whiplash.

First author (year) (origin) [ref]	Design, sample size/conditions*	Interventions (regimen)	Primary outcome measures	Intergroup difference	Effect size (Cohen's *d*)	Adverse event	Author's conclusion
Kwak (2012) (Korea) [23]	Assessor blind RCT, 40 patients with WAD (n.r.)	(A) AT plus UC (6 sessions for 2 weeks, 15 min for one session, *n* = 20) (B) UC (PT, exercise, and sufficient rest, *n* = 20)	(1) VAS (P)	(1) Sig. (*P* < 0.001)	Insufficient data	Three mild reactions (two with mild bruising, one with fatigue) and no serious adverse reactions	AT was associated with a significant alleviation of pain

Tobbackx (2013) (Belgium) [24]	Assessor blind RCT (cross-over), 39/38 patients with chronic WAD (grades I–III)	(A) AT (1 session, 20 min, *n* = 20) (B) Relaxation (1 session, 20 min, *n* = 18)	(1) Pain sensitivity CPM trapezius (2) Pain sensitivity CPM calf	(1) Sig. ( *P* < 0.001) (2) Sig. (*P* < 0.001)	(1) −0.6 (2) −0.1	n.r.	One session of AT results in acute improvements in pressure pain sensitivity in the neck and calf of patients with chronic WAD. AT had no effect on CPM or temporal summation of pressure pain

Cameron (2011) (Australia) [25]	Patient blind RCT, 244/116 patients with chronic or subacute WAD (grade I or II)	(A) EA (12 sessions for six-week period, frequency 2–5 Hz, 1.5 volts, *n* = 52) (B) Sham EA (penetrating, non-acupoint, no electronic stimulation, 12 sessions for six-week period, *n* = 64)	(1) VAS (P) (2) NDI (F) (3) SF-36	(1) Sig. (*P* = 0.05) at 3 months F/U, and (*P* = 0.007) at 6 months F/U (2) NS (*P* > 0.05) (3) NS (*P* > 0.05)	(1) −0.5 (2) −0.4 (3) 0.3	Six mild reactions (slight pain, sweating, and low blood pressure) and no serious adverse reactions	EA was associated with a reduction in pain intensity, but not clinically significant

Han (2011) (Korea) [26]	Patient blind RCT, 58 patients with neck pain due to WAD (n.r.)	(A) EA plus HM (8 sessions for 4 weeks, 15 min for one session, *n* = 29) (B) Sham EA plus HM (penetrating, same acupoints, no electronic stimulation, 8 sessions for 4 weeks, 15 min for one session, *n* = 29)	(1) VAS (P) (2) NDI (F)	(1) Sig. (*P* = 0.043) (2) NS	(1) 0.8 (2) 0.5	n.r.	Cotreatment with EA could be recommended as a useful therapy for WAD patients

Tough (2010) (UK) [27]	Patient blind RCT, 41/34 patients with WAD (grade II)	(A) DN plus PT (1 session per week, total 2–6 sessions, *n* = 17) (B) Sham-DN plus PT (nonpenetrating 2–6 sessions, once a week, *n* = 17)	(1) SF-MPQ (P) (2) m-BPI-sf (P) (3) NDI (F) (4) HAD-A	(1) NS (*P* = 0.67) (2) NS (*P* = 0.56) (3) NS (*P* = 0.43) (4) NS (*P* = 0.63)	(1) 0.2 (2) 0.3 (3) 0.1 (4) −0.03	None	Large RCT is both feasible and clinically relevant

Aigner (1998) (Austria) [28]	RCT, 61 patients with WAD (grades I or II)	(A) AT (2–8 sessions, n.r. for treatment duration, *n* = 28) (B) Drugs (chlormezanone and paracetamol) plus PT (*n* = 33)	(1) ROM (F) (2) duration of acute complaints and drug intake	n.r.	Insufficient data	n.r.	AT can improve ROM and reduce the duration of acute complaints and drug intake

AT: acupuncture; CMI: Cornell medical index; CPM: conditioned pain modulation; DN: dry needling; EA: electro-AT; F: function; F/U: follow-up; HAD-A: hospital anxiety and depression score anxiety subscale; HM: herbal medicine; m-BPI-sf: modified brief pain inventory short-form; N/A: not applicable; NDI: neck disability index; n.r.: not reported; NS: not significant; P: pain intensity; PT: physiotherapy; RCT: randomised clinical trial; ROM: range of motion; SDS: self-rating depression scale; SF-MPQ: short-form McGill pain questionnaire; SF-36: short-form health questionnaire; Sig.: significant; UC: usual care; VAS: visual analogue scale; WAD: whiplash associated disorder.

*Condition is based on Quebec Task Force Classification of WAD.

**Table 2:** Details of the treatment regimen.

First author (year) [ref]	Treatment acupoints	Stimulation technique	Total treatment (session)	Duration of the trials	Timing of the primary endpoint collection
Kwak (2012) [23]	(A) Flexible selection considering the painful lesion (SI2, SI3, SI5, SI7, LI 11, SI 15, SI 14, BL10, BL12, BL13, BL14, BL60, BL62, BL66, GB20, GB21, GB40, GB41, TE15, TE5)	Rotating needles using the index finger and thumb after insertion to a 1.0–2.0 cm depth using a guide tube	6	2 weeks	2 weeks

Tobbackx (2013) [24]	(A) Individually tailored selection of the following points: GV14, C1–C7, GB20, SI11, GB21, TE15, SI14, BL17, SP10, SI3, BL64, TE5, GB41, Ear Zero point, Ear Jerome point, Ear C0	n.r.	1	1 day	1 day

Cameron (2011) [25]	(A) GB39, GB20, LI14, SI6 bilaterally (B) Acupoints 20–30 mm away from (A)	Electrical	12	6 weeks	6 months

Han (2011) [26]	(A), (B) BL10, GB20, GB21, SI14, SI15, SI11	Electrical	8	4 weeks	8 weeks

Tough (2010) [27]	Myofascial trigger points (MTrPs) MTrPs were defined as “tender muscle points (which occur with or without a taut band) and which on sustained palpation (up to 10 seconds) reproduce the patient's pain	Sparrow pecking motion (moving up and down five or six times)	2–6	2–6 weeks	6 weeks

Aigner (1998) [28]	TB5, SI6 bilaterally	n.r.	n.r.	n.r.	n.r.

n.r.: not reported.

## References

[1] Spitzer WO, Skovron ML, Salmi LR (1995). Scientific monograph of the Quebec Task Force on whiplash-associated disorders: redefining ’Whiplash’ and its management. *Spine*.

[2] Eck JC, Hodges SD, Humphreys SC (2001). Whiplash: a review of a commonly misunderstood injury. *American Journal of Medicine*.

[3] Cassidy JD, Linda JC, Coté P, Lemstra M, Berglund A, Nygren Å (2000). Effect of eliminating compensation for pain and suffering on the outcome of insurance claims for whiplash injury. *New England Journal of Medicine*.

[4] Galasko CSB, Murray P, Stephenson W (2002). Incidence of whiplash-associated disorder. *BC Medical Journal*.

[5] Rushton A, Wright C, Heneghan N, Eveleigh G, Calvert M, Freemantle N (2011). Physiotherapy rehabilitation for whiplash associated disorder II: a systematic review and meta-analysis of randomised controlled trials. *British Medical Journal*.

[6] Ernst E (2006). Acupuncture—a critical analysis. *Journal of Internal Medicine*.

[7] Ezzo J, Berman B, Hadhazy VA, Jadad AR, Lao L, Singh BB (2000). Is acupuncture effective for the treatment of chronic pain? A systematic review. *Pain*.

[8] Ernst E, Pittler MH, Wider B (2006). *The Desktop Guide to Complementary and Alternative Medicine: An Evidence Based Approach*.

[9] Mayor DF (2006). *Electroacupuncture: A Practical Manual and Resource*.

[10] Furlan AD, van Tulder MW, Cherkin DC (2005). Acupuncture and dry-needling for low back pain. *Cochrane Database of Systematic Reviews*.

[11] Scholten-Peeters GGM, Bekkering GE, Verhagen AP (2002). Clinical practice guideline for the physiotherapy of patients with whiplash-associated disorders. *Spine*.

[12] South Australian Centre for Trauma and Injury Recovery for the Motor Accident Commission Clinical guidelines for best practice management of acute and chronic whiplash-associated disorders. http://www.nhmrc.gov.au/guidelines/publications/cp112.

[13] Binder AI (2008). Neck pain. *Clinical Evidence*.

[14] Fu L-M, Li J-T, Wu W-S (2009). Randomized controlled trials of acupuncture for neck pain: systematic review and meta-analysis. *Journal of Alternative and Complementary Medicine*.

[15] Furlan AD, Yazdi F, Tsertsvadze A (2012). A systematic review and meta-analysis of efficacy, cost-effectiveness, and safety of selected complementary and alternative medicine for neck and low-back pain. *Evidence-Based Complementary and Alternative Medicine*.

[16] Trinh KV, Graham N, Gross AR (2006). Acupuncture for neck disorders. *Cochrane Database of Systematic Reviews*.

[17] Vickers AJ, Cronin AM, Maschino AC (2012). Acupuncture for chronic pain: individual patient data meta-analysis. *Archives of Internal Medicine*.

[18] White AR, Ernst E (1999). A systematic review of randomized controlled trials of acupuncture for neck pain. *Rheumatology*.

[19] Moore A, Jackson A, Jordon J Clinical guielines for the physiotherapy management of whiplash associated disorder (WAD).

[20] TRACsa: Trauma and Injury Recovery Clinical guideline for best practice managmenet of acute and chronic whiplash associated disorders: clinical resource guide.

[21] International Chiopractors Association of Califonia Management of whiplash associated disorders.

[22] Motor Accidents Authority Guidelines for the management of acute whiplash-associated disorders for health professionals.

[23] Kwak H-Y, Kim J-I, Park J-M (2012). Acupuncture for Whiplash-associated disorder: a randomized, waiting-list controlled, pilot trial. *European Journal of Integrative Medicine*.

[24] Tobbackx Y, Meeus M, Wauters L (2013). Does acupuncture activate endogenous analgesia in chronic whiplash-associated disorders? A randomized crossover trial. *European Journal of Pain*.

[25] Cameron ID, Wang E, Sindhusake D (2011). A randomized trial comparing acupuncture and simulated acupuncture for subacute and chronic whiplash. *Spine*.

[26] Han SY, Lee JY, Park SH (2011). A clinical study on effect of electro-acupuncture treatment for whiplash injury patients caused by traffic accident. *Journal of Korean Acupuncture & Moxibustion Medicine Society*.

[27] Tough EA, White AR, Richards SH, Campbell JL (2010). Myofascial trigger point needling for whiplash associated pain—a feasibility study. *Manual Therapy*.

[28] Aigner N, Fialka C, Stocker R, SKrbensky G, Vecsei V (1998). Whiplash injuries—a prospective, randomised comparison of conventional therapy and acupuncture. *Osteosynthese International*.

[29] Higgins JPT, Altman DG, Sterne JAC, Higgins JPT, Green S (2011). Chapter 8: assessing risk of bias in included studies. *Cochrane Handbook for Systematic Reviews of Interventions Version 510*.

[30] Schulz KF (2001). Assessing allocation concealment and blinding in randomised controlled trials: why bother?. *Evidence-Based Nursing*.

[31] Fink M, Gutenbrunner C, Rollnik J, Karst M (2001). Credibility of a newly designed placebo needle for clinical trials in acupuncture research. *Forschende Komplementarmedizin und Klassische Naturheilkunde*.

[32] Moffet HH (2009). Sham acupuncture may be as efficacious as true acupuncture: a systematic review of clinical trials. *Journal of Alternative and Complementary Medicine*.

[33] MacPherson H, Altman DG, Hammerschlag R (2010). Revised standards for reporting interventions in clinical trials of acupuncture (STRICTA): extending the consort statement. *Acupuncture in Medicine*.

[34] Hammerschlag R, Milley R, Colbert A (2011). Randomized controlled trials of acupuncture (1997–2007): an assessment of reporting quality with a CONSORT—and STRICTA-based instrument. *Evidence-Based Complementary and Alternative Medicine*.

[35] Côté P, Cassidy JD, Carroll L, Frank JW, Bombardier C (2001). A systematic review of the prognosis of acute whiplash and a new conceptual framework to synthesize the literature. *Spine*.

[36] Côté P, Hogg-Johnson S, Cassidy JD, Carroll L, Frank JW, Bombardier C (2005). Initial patterns of clinical care and recovery from whiplash injuries: a population-based cohort study. *Archives of Internal Medicine*.

[37] Su T-F, Zhang L-H, Peng M (2011). Cannabinoid CB2 receptors contribute to upregulation of beta-endorphin in inflamed skin tissues by electroacupuncture. *Molecular Pain*.

[38] Su TF, Zhao YQ, Zhang LH (2012). Electroacupuncture reduces the expression of proinflammatory cytokines in inflamed skin tissues through activation of cannabinoid CB2 receptors. *European Journal of Pain*.

